# Knockdown of TMEM30A in renal tubular epithelial cells leads to reduced glucose absorption

**DOI:** 10.1186/s12882-023-03299-8

**Published:** 2023-08-23

**Authors:** Sipei Chen, Xinrou Song, Qiong Xiao, Li Wang, Xianjun Zhu, Yang Zou, Guisen Li

**Affiliations:** 1 Department of Nephrology, Sichuan Provincial People’s Hospital, School of Medicine, University of Electronic Science and Technology of China, No. 32, West 2Nd Duan, 1St Circle Road, Qingyang District, Chengdu, 610072 Sichuan China; 2 Department of Nephrology, Chengdu Fifth People’s Hospital, Chengdu, China

**Keywords:** SGLT2, TMEM30A, Renal tubular epithelial cells

## Abstract

The kidney reabsorbs large amounts of glucose through Na^+^-glucose cotransporter 2 (SGLT2). P4-ATPase acts together with the β-subunit TMEM30A to mediate the asymmetric distribution of phosphatidylserine (PS), phosphatidylethanolamine (PE), and other amino phospholipids, promoting plasma membrane and internal vesicle fusion, and facilitating vesicle protein transport. We observed reduced TMEM30A expression in renal tubules of DKD and IgA patients, suggesting a potential role of TMEM30A in renal tubular cells. To investigate the role of TMEM30A in renal tubules, we constructed a TMEM30A knockdown cell model by transfecting mouse kidney tubular epithelium cells (TCMK-1) with TMEM30A shRNA. Knockdown of TMEM30A in TCMK-1 cells attenuated vesicle transporter protein synthesis, resulting in reduced transport and expression of SGLT2, which in turn reduced glucose absorption. These data suggested that TMEM30A plays a crucial role in renal tubules.

**Supplementary Information:**

The online version contains supplementary material available at 10.1186/s12882-023-03299-8.

## Introduction

Blood glucose is freely filtered by renal glomeruli and, in physiological condition, completely reabsorbed in proximal convoluted tubules [1]. In addition, the kidney also produces new glucose through gluconeogenesis, so as to maintain blood glucose level and overall metabolic homeostasis [2]. More than 90% of the glucose filtered through the glomerulus is reabsorbed by Na^+^-glucose cotransporter 2 (SGLT2) located on the brush border of S1 and S2 segments of the proximal tubule [3].

SGLT2 binds glucose and sodium equimolarly. Glucose enters cells along the concentration gradient of Na^+^, and then is transported to the surrounding capillary network through SGLT2 on the basement membrane side of epithelial cells, so as to complete its reabsorption. The remaining less than 10% is reabsorbed by SGLT1 located in the S2/S3 segment of the proximal tubule [4, 5]. SGLT2 protein, encoded by SLC5A2, consists of 672 amino acids and is a low-affinity, high-volume glucose transporter protein [6–11].

Eukaryotic biofilm consists of phospholipid bilayers with asymmetric distribution of phospholipids on both sides, where phosphatidylserine (PS) and phosphatidylethanolamine (PE) are mainly concentrated in the inner lobe of the eukaryotic biofilm, while sphingomyelin (SM) is almost always located in the outer lobe of the membrane, and phosphatidylcholine (PC) is uniformly distributed on both sides of the membrane and requires maintenance by flippases, floppases and scramblases [12]. The asymmetric distribution of phospholipids on biofilm not only plays a protective role and enhances intercellular signal transduction but also promotes the fusion of plasma membrane and internal vesicles, which facilitates vesicle protein transport [13]. P4-ATPase is a phospholipid invertase whose main function is to mediate the turning of phosphatidyl phospholipids such as PS and PE from the outer side of the cell membrane to the inner side of the cell membrane or from the inner membrane of the organelle to the cytoplasmic side [14–16]. The Atp10a-deficient mice in the P4-ATPase family have hyperinsulinemia, insulin resistance, and altered responses to insulin stimulation in peripheral tissues [17]. P4-ATPase is a heterodimer that requires the β-subunit for its physiological function. P4-ATPase and the CDC50 (TMEM30) family cooperate in the formation and trafficking of transport vesicles between late Golgi and early endosomes [18]. CDC50 family is a class of highly conserved transmembrane proteins, with molecular weight of 50–60 kDa [19]. There are three family members TMEM30A, B, and C. TMEM30A and TMEM30B are widely expressed in humans, while TMEM30C is only expressed in testis and brain [20, 21]. It has been shown that knockdown of TMEM30A in cell lines disrupts the endoplasmic reticulum outlet of P4-ATPase, leading to the formation of membrane folds and thus inhibiting cell migration. Due to exposure to PS in cytoplasmic leaflets, TMEM30A-deficient cells are phagocytosed by macrophages [22]. TMEM30A protein also promotes the uptake of anticancer drugs and choline phospholipids by mammalian cells [23]. TMEM30A is essential for the survival of retinal photoreceptor cells as well as bipolar cells [24]. In the kidney, the podocyte-specific TMEM30A knockout mouse model showed albuminuria, podocyte degeneration, the proliferation of thylakoid cells with significant extracellular matrix accumulation, and eventual progression to focal segmental glomerulosclerosis [25]. Taken together, we hypothesized that TMEM30A play an important role in the kidney.

We observed a significant reduction in renal tubular TMEM30A expression in DN and IgA patients, suggesting that TMEM30A may have an important function in the renal tubules. In this study, TMEM30A knockdown model of renal tubular epithelial cells was established in vitro. Afterwards, the effects of knockdown of TMEM30A on SGLT2 and glucose reabsorption in renal tubular epithelial cells were investigated by using a series of molecular biology and cell biology methods, in order to clarify the role of TMEM30A in renal tubules.

## Methods

### Patients

Patients with available renal biopsy specimens diagnosed at the Sichuan Provincial People's hospital were included in this study. 6 cases each of normal control (normal kidney tissue next to cancer), diabetic kidney disease (DKD) and IgA Nephropathy (IgA) renal tissue were collected, and none of them used SGLT2 inhibitor. This study was approved by the ethics committee of the Sichuan Provincial People's Hospital (No. 2020–224). Informed consent was obtained from all participants, and the privacy rights of human subjects were always protected. All methods were performed in accordance with the relevant guidelines and regulations.

### Immunohistochemistry

The 4 μm paraffin sections were deparaffinized in water. After the slices were placed in an appropriate amount of sodium citrate antigen retrieval solution for antigen retrieval, endogenous peroxidase was blocked. The sections were incubated overnight with primary antibodies: TMEM30A (1:200, bs-16576R, BIOSS) and SGLT2 (1:500, ab85626, Abcam). Sections were then incubated with horseradish peroxidase-conjugated secondary antibodies, developed with DAB chromogenic solution, counterstained with hematoxylin, differentiated, and returned to blue. The sections were observed under light microscope.

### Cell culture and lentivirus transfection

Mouse kidney tubular epithelium cells (TCMK-1, ATCC CCL-139™) were purchased from American Type Culture Collection and cultured in Eagle’s Minimum Essential Medium (EMEM) supplemented with 10% fetal bovine serum (FBS) and penicillin/streptomycin (Sigma-Aldrich) in a humidified atmosphere of 5% CO_2_ at 37 ℃.

Lentiviral vectors with TMEM30A shRNA or enhanced green fluorescent protein (GFP) were constructed. Lentiviral vector (GV248) and packaging vectors were transfected into the TCMK-1 cells using the transfection reagent RNAi-mate (Genepharma, Shanghai, China). The media containing lentiviruses were discarded 72 h later, and incubated with puromycin. Over-expression efficiency was detected two weeks after screen.

### Quantitative PCR

Total RNA was extracted using TRIzol reagent according to the manufacturer’s instructions (Invitrogen, USA). Reverse-transcription of the extracted RNA was performed using PrimeScript RT reagent kit with gDNA Eraser (Perfect Real Time, TaKaRa, #RR820A). Quantitative PCR was performed on the CFX96 Real-time PCR Detection System (Bio-Rad) using TB Green Premix Ex Taq II (Tli RNaseH Plus, TaKaRa, #RR820A). Relative mRNA expression levels were calculated using the 2^−ΔΔCt^ method and normalized to the housekeeping gene *GAPDH*. The primers used for quantitative PCR are as follows: *TMEM30A* gene (mouse, Forward primer: TGAAGACACCACAATTGCGC and Reverse primer: TCGAGGCTCTTTTCCAGG), *Rab6* gene (mouse, Forward primer: TCGGAGGTCCTGAAAGGAA and Reverse primer: TGTTTCCGGGAAGTCGT) *SGLT2* gene (mouse, Forward primer: TTCCTGCTGGTCATTGGTGGTT and Reverse primer: CCAGGAAGTAGTAGCCAACT), and *GAPDH* internal reference gene (mouse, Forward primer: AGGTCGGTGAACGGATTTG and Reverse primer: GGGGGTCGTTGATGGCAACA).

### Western blotting

The collected cells were lysed in RAPI lysis buffer (1% PMSF, Beyotime, China) on ice for 30 min, followed by centrifugation at 15,000 rpm for 10 min. Lording Buffer (Beyotime, China) was added at 95°C for 5 min. All protein concentrations were determined using the BCA protein assay reagents (Beyotime, China). Equal amounts of protein were loaded and separated on SDS-PAGE gels, and then transferred to PVDF membranes. The membranes were blocked in 5% BSA and incubated at 37 C for 1 h. Then the membranes were incubated with primary antibodies overnight at 4 C, including TMEM30A (1:1000, bs-16576r, BIOSS, China), SGLT2 (1:1000, ab37296, Abcam, Britain), Rab6 (1:1000, ab954, Abcam, Britain), and GLUT2 (1:1000, pa5-97263, Invitrogen, America). Corresponding HRP-conjugated secondary antibodies (ZenBio, China) were added at a ratio of 1:5000. PVDF membranes were observed by ECL ultra-sensitive chemiluminescence (ECL, ZenBio, China). The signal intensity of the blots was quantified using Image J software.

### Immunocytochemistry

Cells were seeded in glass bottom cell culture dishes at a density of 3000 cells/cm^2^. Then the cells were incubated with primary antibodies overnight at 4 °C, including TMEM30A (1:200, bs-16576r, BIOSS, China), SGLT2 (1:200, sc-393350, Santa Cruz Biotechnology, America), and Rab6 (1:200, ab954, Abcam, Britain). Thereafter, the cells were incubated with labeled fluorescent secondary antibodies Alexa flow 647 (1:300, 550048, ZenBio, China), and TRITC (1:300, 511102, ZenBio, China). Nuclei were stained with DAPI (ZenBio, China). Imaging was performed using confocal microscope, with a 63X objective (Leica, Germany). The positive areas (%) for TMEM30A were measured in kidney tubules by Image J software [26, 27].

### Glucose uptake

Cells were incubated in 24-well plates for 24 h and then starved in glucose-free medium for 24 h. Thereafter, 5, 10, 22, and 33 mmol/L concentrations of glucose were added for 24, 48, and 72 h, respectively. The glucose content in the medium was measured according to the glucose content determination kit (Solarbio, BC2505).

### Statistical analysis

All data were statistically analyzed by SPSS 25.0 software, and the results of normally distributed measures were expressed as x ± SD. One-way ANOVA was used to compare the differences between groups, an independent samples t-test was used to compare the differences between two groups, and *P* < 0.05 was considered statistically significant.

## Results

### Expression of TMEM30A and SGLT2 in human kidney

**Fig. 1:**
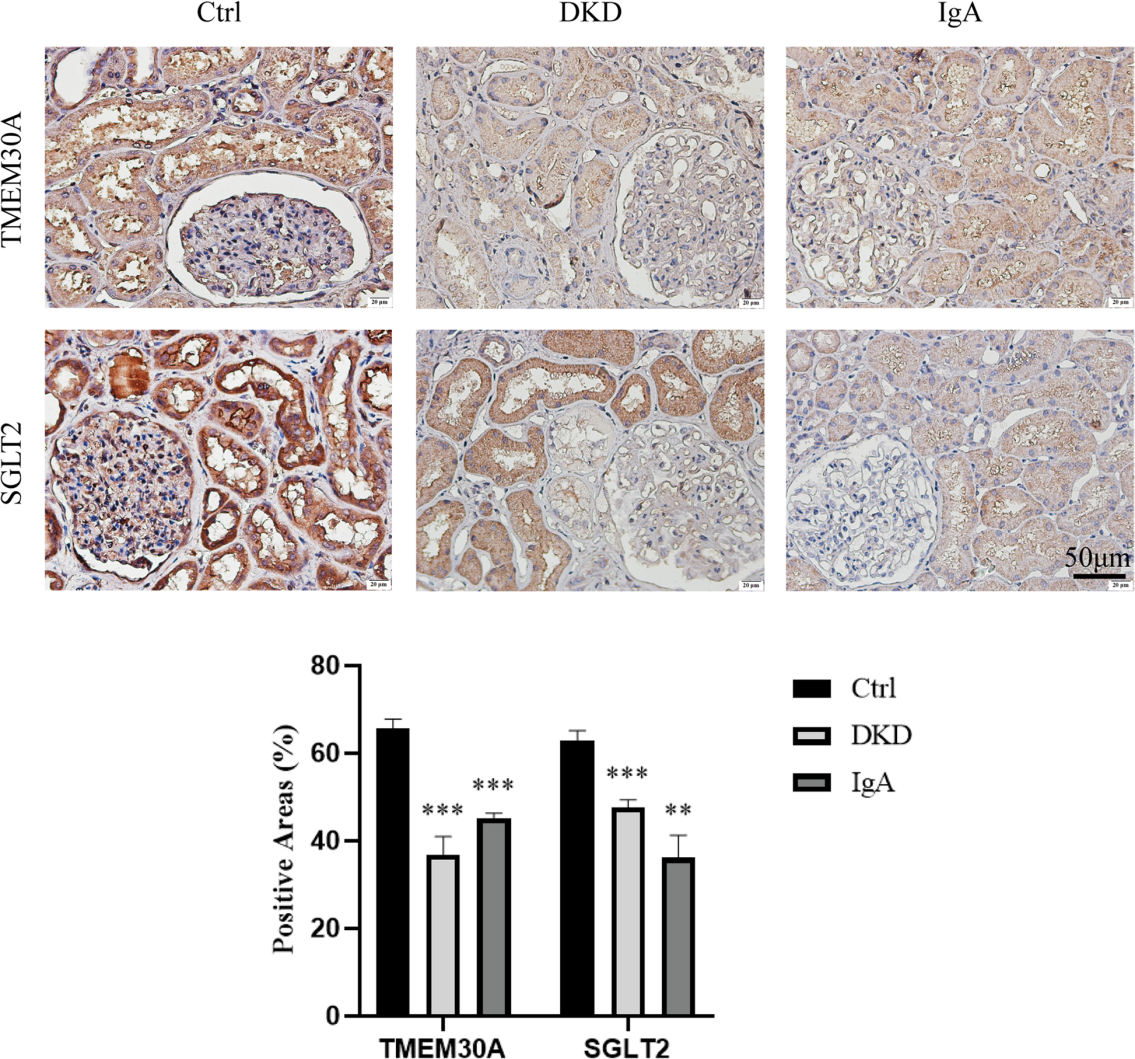
Expression of TMEM30A and SGLT2 in the tubules was observed in the kidney tissues of normal controls, DKD, and IgA groups. Immunohistochemical staining of TMEM30A and SGLT2 (bar = 50 μm). Data are expressed as x ± SD (*n* = 6). Compared with the Ctrl group by t-test, *P < 0.05, **P < 0.01, ***P < 0.001

Immunohistochemical staining of renal tissues from normal, DKD and IgA patients revealed that TMEM30A expression was reduced in the renal tubules of DKD and IgA patients compared to normal subjects (Fig. 1). Moreover, SGLT2 expression also appeared to be reduced (Fig. 1), possibly due to negative feedback regulation or the effect of gluconeogenesis. The results suggested a possible association between TMEM30A and SGLT2.

### TMEM30A knockdown lentivirus selection in TCMK-1 cells

**Fig. 2:**
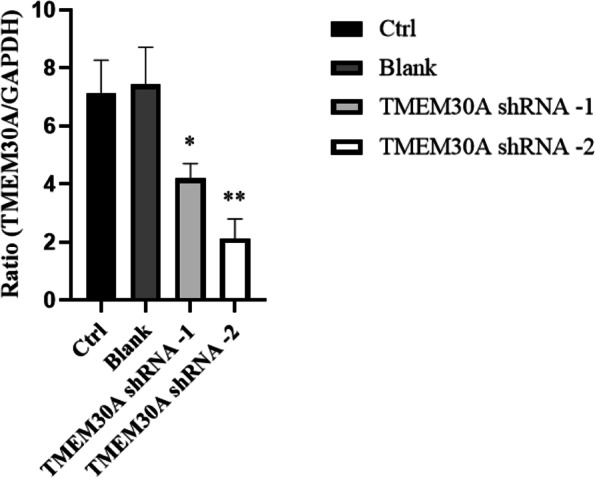
Expression of TMEM30A mRNA in TCMK-1 cells transfected with two TMEM30A shRNAs. TCMK-1 cells were transfected with two TMEM30A shRNAs. And TMEM30A mRNA expression was determined by qPCR. Data are expressed as x ± SD (*n* = 3). Compared with the Ctrl group by t-test, *P < 0.05, **P < 0.01, ***P < 0.001

TCMK-1 cells were transfected with two TMEM30A shRNAs and puromycin screened, and their RNA was extracted and reverse-transcribed into cDNA. The mRNA expression levels were detected by qPCR. The results showed that the TMEM30A mRNA expression in the two groups transfected with different TMEM30A shRNAs were significantly lower than that in the normal cell group and the empty vector group, but the expression of TMEM30A-shRNA-2 group was the lowest (Fig. 2). Therefore, TMEM30A shRNA-2 was selected for transfection.

### Effects of TMEM30A knockdown on TCMK-1 cell-associated proteins

**Fig. 3:**
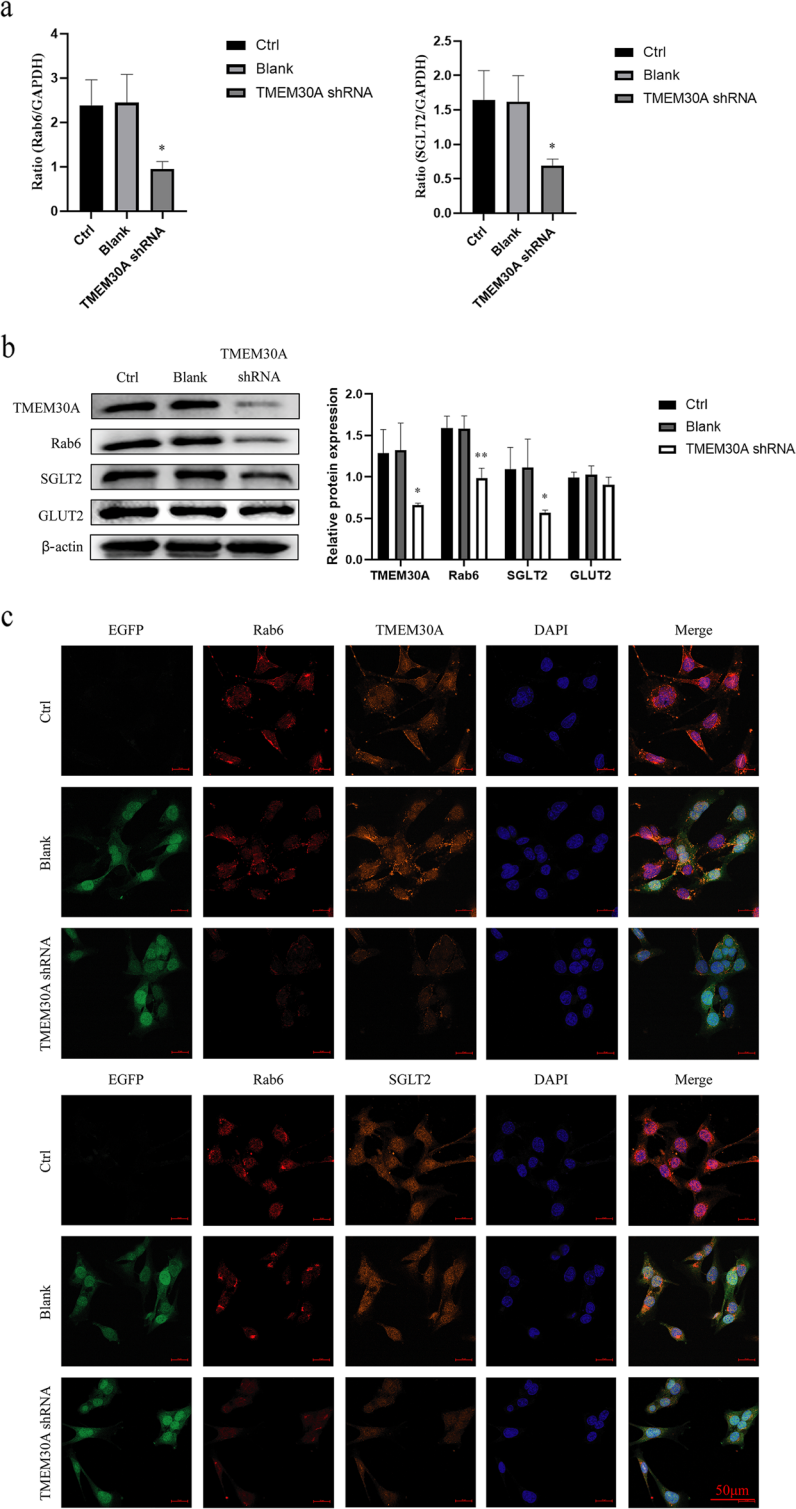
Effects of TMEM30A knockdown on TCMK-1 cells associated proteins. **a** TMEM30A, Rab6, and SGLT2 mRNA expressions were determined by qPCR in TCMK-1 cells transfected with TMEM30A shRNAs. **b** Protein expressions of TMEM30A, Rab6, SGLT2, and GLUT2 in TCMK-1 cells were detected by western blotting. **c** Immunofluorescent analysis with nuclei stained with DAPI (blue), EGFP (green), Rab6 (red), TMEM30A and SGLT2 (orange) in TCMK-1 cells (bar = 50 μm). Data are expressed as x ± SD (*n* = 3). Compared with the Ctrl group by t-test, *P < *0.05*, **P < 0.01, ***P < 0.001

The qPCR, Western blot, and immunocytochemical staining analysis of TCMK-1 cells with TMEM30A knockdown showed that the expression of SGLT2 and vesicle transporter protein Rab6 were significantly decreased in the TMEM30A shRNA group compared with normal cells and the empty vector group (Fig. 3). The results indicated that TMEM30A knockdown could affect the transport and expression of SGLT2 by decreasing the expression of vesicular transporter protein Rab6 and thus the expression of SGLT2. However, there was no significant decrease in GLUT2 expression in the TMEM30A shRNA group.

### Effects of TMEM30A knockdown on glucose uptake in TCMK-1 cells

**Fig. 4:**
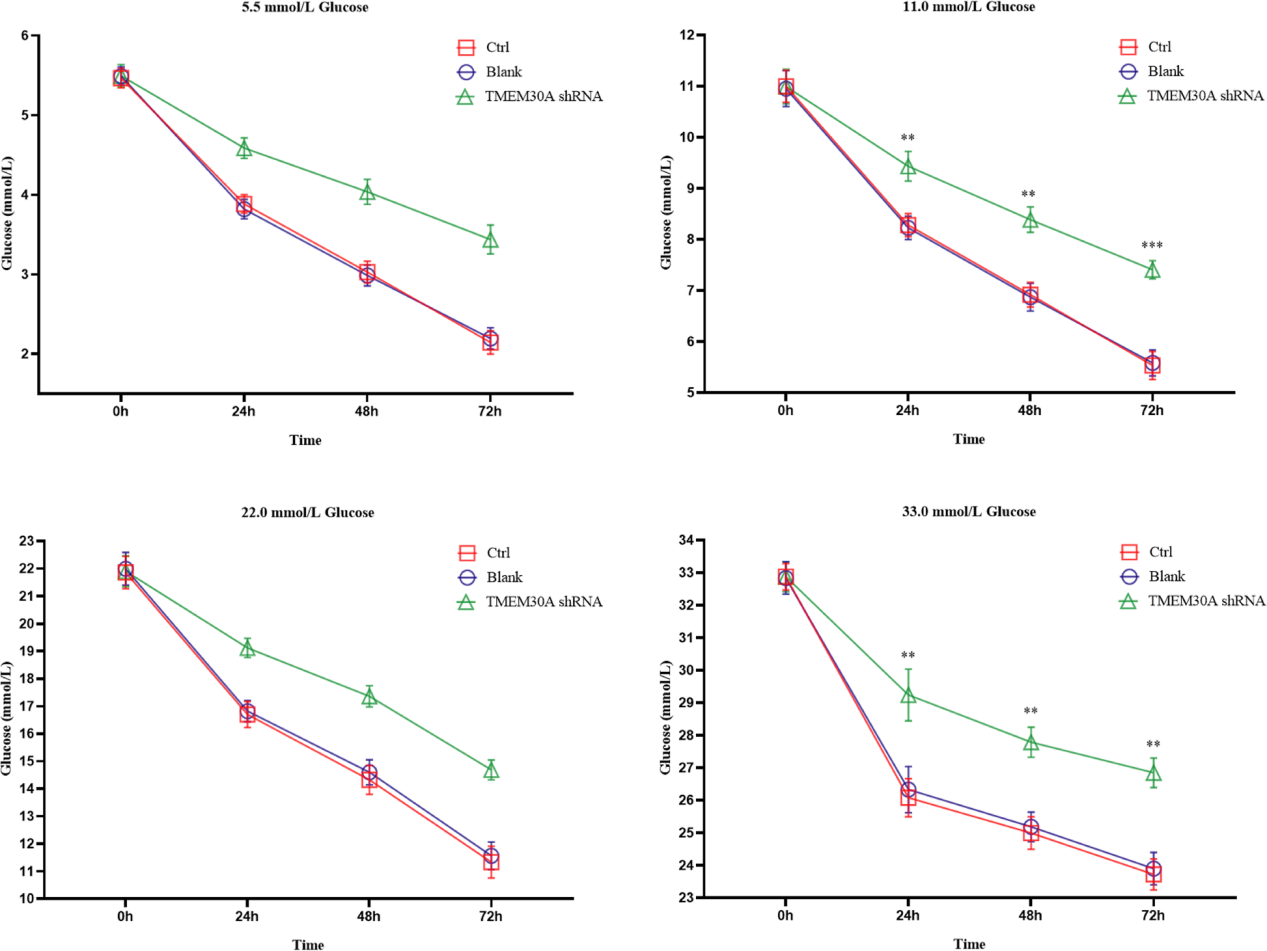
Effects of TMEM30A knockdown on glucose uptake by TCMK-1 cells. The glucose content remaining in the culture medium after TCMK-1 cells were treated with different concentrations of glucose for different time points were detected by a glucose detection kit. Data are expressed as x ± SD (*n* = 3). Compared with the Ctrl group by t-test, *P < 0.05, **P < 0.01, ***P < 0.001

TCMK-1 cells were treated with different concentrations of glucose for different time points, and the remaining glucose content in the medium was examined. The addition of 5, 10, and 22 mmol/L glucose resulted in higher residual glucose content in the TMEM30A shRNA group compared to the normal cells and the empty vector group (Fig. 4), indicating that glucose uptake was less in this group, suggesting that glucose uptake was affected by the knockdown of TMEM30A. Meanwhile, after adding 33 mmol/L glucose to the culture, the glucose uptake rates of all three groups of cells at 48 h and 72 h were significantly inhibited (Fig. 4), probably because high glucose caused extensive cell death.

## Discussion

The kidney has an important regulatory role in maintaining systemic glucose homeostasis [8]. The main role is played by SGLT2 localized at the brush border of the proximal tubule in the S1 and S2 segments of the kidney [9, 11]. TMEM30A, the β-subunit of P4-ATPase, is important for maintaining the asymmetric distribution of phospholipids, stability of cell membrane structure, vesicular protein transport, blood coagulation, regulation of membrane protein function, recognition of apoptosis, cell differentiation, and establishing cell polarity. It can protect the endoplasmic reticulum to form vesicles, ensure vesicle transport and promote vesicle protein transport [12, 19]. It has been shown that podocyte-specific TMEM30A knockout mouse models develop podocyte degeneration leading to their damage, thylakoid cell proliferation, and extracellular matrix accumulation, which in turn cause albuminuria and eventually progress to focal segmental glomerulosclerosis [25]. This study reported reduced expression of TMEM30A in renal tubules of kidney sections from DKD and IgA patients, indicating the importance of TMEM30A in renal tubular epithelial cells. As a β-subunit of PS flippase, TMEM30A may affect the transport of essential proteins. By constructing a TMEM30A knockdown model in renal tubular epithelial cells, we demonstrated that TMEM30A plays a crucial role in maintaining renal tubular function. After TMEM30A knockdown, both mRNA and protein levels of SGLT2 were significantly reduced, resulting in reduced glucose reabsorption. Moreover, the loss of TMEM30A resulted in reduced uptake of glucose upon administration of low and moderate concentrations of glucose. However, after administration of a high concentration of glucose, the absorption of glucose was significantly reduced, indicating that a high concentration of glucose caused cell damage and even death.

As a secreted protein, SGLT2 needs to travel from the endoplasmic reticulum (ER) through the Golgi complex to the trans Golgi network (TGN), where it will be packaged into a cytoplasmic vehicle for delivery to the plasma membrane or extracellular region. Between the TGN and the plasma membrane, proteins are transported mainly by several different protein transport pathways mediated by specific types of vesicles [28]. Most flippase enzymes in the P4-ATPase family are composed of a catalytic α subunit and a non-catalytic β subunit of the CDC50 family of integral membrane proteins, which work together to help with the transition from the Golgi and endosomes membrane sprouted vesicular proteins, thereby affecting the sorting and localization of many different proteins in secretory and endocytic pathways [20]. Ras-associated protein 6 (Rab6) is a kind of small GTPases involved in vesicular transport during secretion and endocytosis. Rab6 locates in Golgi apparatus and TGN membrane, and plays an important role in the formation and transport of vesicles [29–31]. Rab6 is involved in the composition of Golgi apparatus and cytoplasmic vesicles, regulates the transport of Golgi apparatus, and maintains its integrity and stability. Rab6 controls both anterograde and retrograde transport, from and toward the Golgi apparatus. It is not only involved in regulate Golgi-to-ER transport, but also involved in anterograde trafficking from the Golgi apparatus to the plasma membrane [32, 33]. In addition, Rab6 and its effector protein are required for the regulation of vesicle fission from Golgi apparatus [34]. Therefore, we studied the formation and transport of vesicles and Golgi transport by detecting the expression of Rab6 in renal tubular epithelial cells. The results showed that after the loss of TMEM30A, the mRNA and protein levels of Rab6 were significantly reduced, and the expression was down-regulated, indicating that knockdown of TMEM30A affects the synthesis of vesicular transporters. These data suggested that the reduction of TMEM30A may reduce the interaction with P4-ATPase, which in turn affects its PE flippase activity, resulting in blocked Golgi vesicle formation and reduced trafficking of secreted proteins.

In summary, this study revealed an important role of TMEM30A in renal tubular reabsorption. Knockdown of TMEM30A in renal tubular epithelial cells affects the synthesis of vesicular transport proteins, which in turn affects the transport function of vesicles and causes a decrease in SGLT2 transport and expression, ultimately leading to a decrease in glucose uptake. TMEM30A can affect Golgi function, which in turn causes alterations in SGLT2 expression and transport. This provides another unique perspective for understanding the mechanism of renal tubular reabsorption. Further studies are needed to elucidate the molecular signaling pathway of renal tubular reabsorption after TMEM30A deletion.

### Supplementary Information

**Additional file 1.**

## Data Availability

All datasets generated during this study are included in this published article and its supplementary information files.
