# Supplementary figures and images for: Knockdown of TMEM30A in renal tubular epithelial cells leads to reduced glucose absorption
Source: BMC Nephrol. 2023 Aug 23;24:250. doi: 10.1186/s12882-023-03299-8 (PMC10464243; doi:10.1186/s12882-023-03299-8)

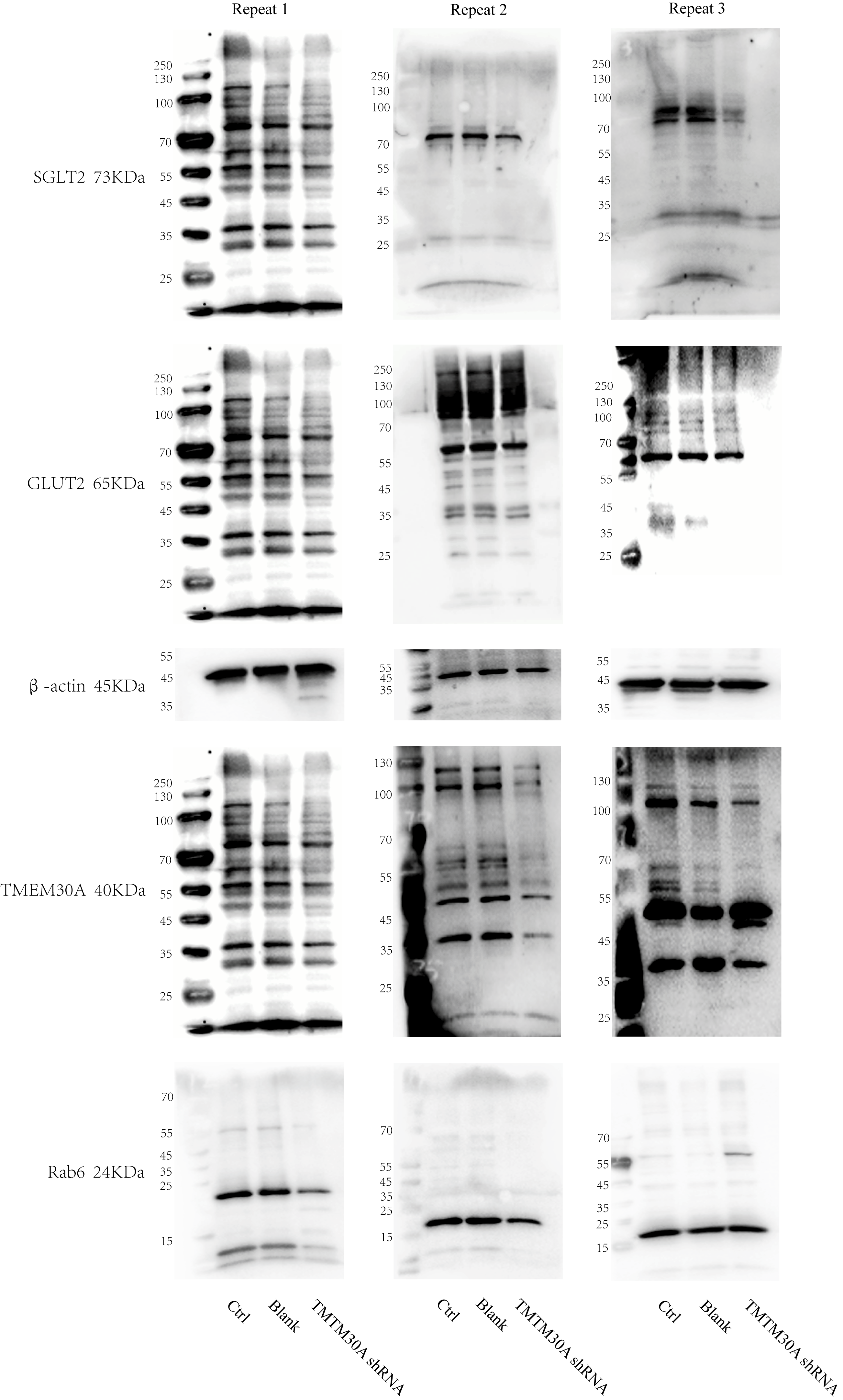

Supplement: Supplementary file 1 — Additional file 1. [file 12882_2023_3299_MOESM1_ESM.tif]
